# Modelling of Granular Fracture in Polycrystalline Materials Using Ordinary State-Based Peridynamics

**DOI:** 10.3390/ma9120977

**Published:** 2016-12-02

**Authors:** Ning Zhu, Dennj De Meo, Erkan Oterkus

**Affiliations:** Department of Naval Architecture, Ocean and Marine Engineering, University of Strathclyde, Glasgow G4 0LZ, UK; n.zhu@strath.ac.uk (N.Z.); dennj.demeo@strath.ac.uk (D.D.M.)

**Keywords:** polycrystalline materials, ordinary state-based peridynamics, transgranular fracture, intergranular fracture

## Abstract

An ordinary state-based peridynamic formulation is developed to analyse cubic polycrystalline materials for the first time in the literature. This new approach has the advantage that no constraint condition is imposed on material constants as opposed to bond-based peridynamic theory. The formulation is validated by first considering static analyses and comparing the displacement fields obtained from the finite element method and ordinary state-based peridynamics. Then, dynamic analysis is performed to investigate the effect of grain boundary strength, crystal size, and discretization size on fracture behaviour and fracture morphology.

## 1. Introduction

Polycrystalline materials are widely used in many different industrial applications. Amongst the various existing polycrystalline materials, metals and ceramics are common examples. Polycrystalline materials are composed of individual crystals that have a particular crystal orientation and are separated from neighbouring crystals via grain boundaries. Microscopic features of polycrystalline materials such as crystal orientation, grain boundary strength, etc. may have a significant effect on the overall macroscopic behaviour of the material, especially on the fracturing behaviour of these materials. Therefore, it is essential to analyse this type of material at the microscopic scale. Although experimental approaches can be useful for this purpose [1,2,3], it is not always possible to perform such experiments and they can also be very expensive. On the other hand, numerical approaches can be a very good alternative. There are various numerical studies available in the literature. For the majority of these studies, cohesive zone elements (CZM) [4,5,6], extended finite element methodology (XFEM) [7,8], and boundary element method (BEM) [9,10] were utilized. Although these techniques are widely used and powerful, they may have limitations for some specific cases and conditions. Instead, a new continuum mechanics formulation, peridynamics (PD) [11], can be considered.

As opposed to partial differential equations that traditional approaches are based on, peridynamics utilizes integro-differential equations without containing any spatial derivatives. Hence, these equations are always applicable regardless of discontinuities such as cracks. Peridynamics has been used for the fracture analysis of many different types of materials and material behaviours [12,13,14,15,16,17,18,19]. It has also been applied for the analysis of polycrystalline materials [20,21,22]. However, these studies used either original bond-based formulation (BB) [11] or non-ordinary state-based (NOSB) [23] formulations. Bond-based formulation has limitations on material constants whereas non-ordinary state-based formulations may encounter the zero-energy mode problem. In order to overcome all these issues, an ordinary state-based (OSB) peridynamic formulation [23,24] can be utilized. The numerical solution of peridynamics is generally obtained by using a meshless scheme. Therefore, the formulation does not suffer from issues such as mesh distortion, especially for problems involving large deformations and fractures. There are also other mesh-free methods available in the literature and used for modelling shear bands in metals [25,26,27,28]: concrete fragmentation [29,30,31], dynamic fracture in thin shells [26,32], and fluid–structure interaction [33]. A good example of a meshless approach used for fracture is the cracking particles method (CPM) [29,34,35]. In CPM, the crack path is represented by a set of cracked particles. CPM is especially useful for complex fracture patterns such as crack branching and coalescence. Other promising techniques used for fracture modelling include smoothed particle hydrodynamics [36,37,38], molecular dynamics [39], the discrete element method [40], and the force potential-based particle method [41]. Although the aforementioned approaches may have certain advantages for particular conditions, in this study peridynamics was chosen for modelling granular fracture in polycrystalline materials. Moreover, the ordinary state-based formulation was utilized for the first time in the literature in order to overcome the limitations of bond-based formulation and zero-energy mode problem of non-ordinary state-based theory. After validating the formulation, several demonstration cases are considered to investigate the effect of grain boundary strength, crystal (grain) orientation, and grain size. 

## 2. Ordinary State-Based Peridynamic Formulation for a Cubic Crystal

**Figure 1:**
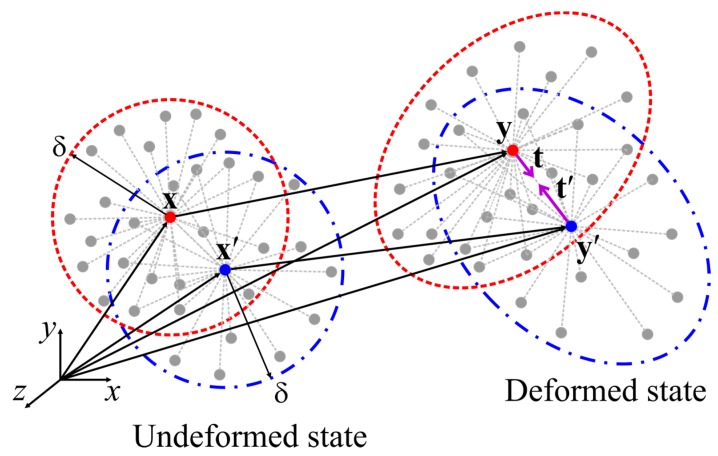
The horizon of the material points located at x and $x^{\prime }$ and the peridynamic forces between them in ordinary state-based peridynamics.

The equation of motion of Ordinary State-Based (OSB) peridynamics (PD) can be written as:

(1) $$\rho (x)\ddot{u}(x,t)=\int_{H}(t(u^{\prime }-u,x^{\prime }-x,t)-t^{\prime }(u^{\prime }-u,x^{\prime }-x,t))dH+b(x,t),$$

where ρ(x) represents the density and $\ddot{u}(x,t)$ is the acceleration of material point x at time t. Moreover, $t(u^{\prime }-u,x^{\prime }-x,t)$ and $t^{\prime }(u^{\prime }-u,x^{\prime }-x,t)$ denote the force density vectors of the material points x and $x^{\prime }$, and $u^{\prime }-u$ represents the difference of displacements of the material points x and $x^{\prime }$ at time t. In Equation (1), *H* represents the peridynamic horizon that defines the range of interaction of a particular material point, as shown in Figure 1. In the peridynamic literature, the size of the horizon is usually represented by the symbol δ.

**Figure 2:**
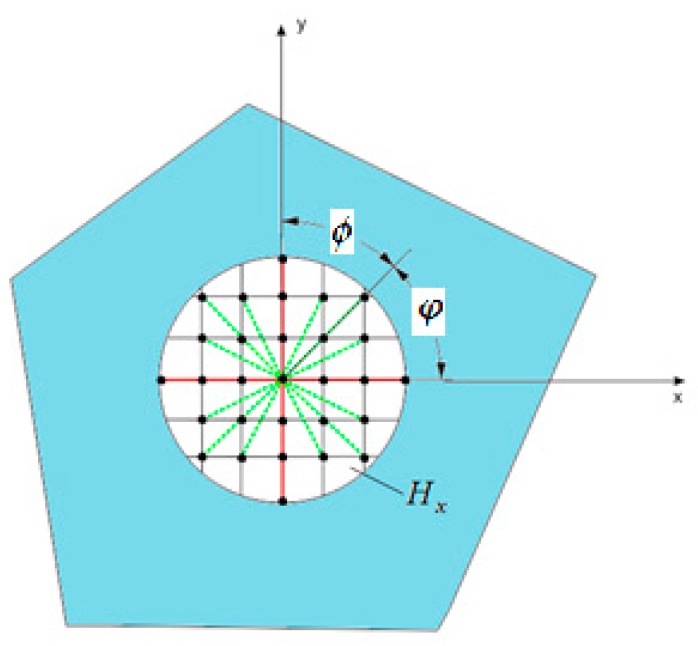
Type 1 bonds (green dashed lines) and Type 2 bonds (red solid lines) for the OSB PD cubic crystal model for a grain orientation of $\varphi =\frac{\pi }{4}$.

Similar to the bond-based (BB) peridynamic model presented in De Meo et al. (2016) [22], the ordinary state-based model for a cubic crystal can be represented using two types of interactions (bonds), as shown in Figure 2.

These are:

1. Type 1 bonds (green dashed lines)—interactions along all directions (ϕ=0~2π),
2. Type 2 bonds (red solid lines)—interactions along the directions of $\phi =\frac{1}{4}\pi ,\frac{3}{4}\pi ,\frac{5}{4}\pi ,\frac{7}{4}\pi$,

where ϕ represents the angle between the orientation of the bond and the crystal (grain) orientation. As an example, bonds within the horizon of a particular material point for a grain orientation of $\varphi =\frac{\pi }{4}$ are shown in Figure 2.

According to OSB PD theory, the strain energy density of a material point can be written as [24]:

(2) $$\begin{array}{l} W_{\left(k\right)}=a\theta_{\left(k\right)}^{2}+b_{T1}\int_{H}\frac{\delta }{\left|x^{\prime }-x\right|}{\left(\left|y^{\prime }-y\right|-\left|x^{\prime }-x\right|\right)}^{2}dH\\ +b_{T2}\sum_{j=1}^{J}\frac{\delta }{\left|x_{\left(j\right)}-x_{\left(k\right)}\right|}{\left(\left|y_{\left(j\right)}-y_{\left(k\right)}\right|-\left|x_{\left(j\right)}-x_{\left(k\right)}\right|\right)}^{2}V_{\left(j\right)} \end{array},$$

where *J* is the total number of material points within the family of material point $x_{\left(k\right)}$.

By using the strain energy density expression given in Equation (2), the peridynamic force densities t and $t^{\prime }$ can be obtained as:

(3) $$t\left(u^{\prime }-u,x^{\prime }-x,t\right)=\frac{1}{2}A\frac{y^{\prime }-y}{\left|y^{\prime }-y\right|},$$

where

(4) $$A=4ad\frac{\delta }{\left|x^{\prime }-x\right|}\Lambda \theta +4\delta \left(b_{T1}+\mu_{T2}b_{T2}\right)s$$

with

(5) $$\mu_{T2}=\{\begin{array}{ccc} 1 & & \text{Type-}2\;\text{bonds}\\ 0 & & \text{otherwise} \end{array}$$

and

(6) $$t^{\prime }\left(u-u^{\prime },x-x^{\prime },t\right)=-\frac{1}{2}B\frac{y^{\prime }-y}{\left|y^{\prime }-y\right|},$$

where

(7) $$B=4ad\frac{\delta }{\left|x^{\prime }-x\right|}\Lambda \theta^{\prime }+4\delta \left(b_{T1}+\mu_{T2}b_{T2}\right)s.$$

In Equations (3) and (6), y and $y^{\prime }$ represent the location of material points x and $x^{\prime }$ after deformation, i.e., y=x+u and $y^{\prime }=x^{\prime }+u^{\prime }$ (see Figure 1). The PD dilatation, θ, for a crystal can be expressed as:

(8) $$\theta_{\left(k\right)}=d\int_{H}\frac{\delta }{\left|x^{\prime }-x\right|}\left(\left|y^{\prime }-y\right|-\left|x^{\prime }-x\right|\right)\Lambda dH,$$

and the parameter, Λ, is defined as:

(9) $$\Lambda =\left(\frac{y^{\prime }-y}{\left|y^{\prime }-y\right|}\right)\cdot \left(\frac{x^{\prime }-x}{\left|x^{\prime }-x\right|}\right).$$

The stretch parameter *s* can be expressed as:

(10) $$s=\frac{\left|y^{\prime }-y\right|-\left|x^{\prime }-x\right|}{\left|x^{\prime }-x\right|}.$$

The PD material parameter *a* is associated with the deformation involving dilatation, $\theta_{\left(k\right)}$. The remaining material parameters, $b_{T1}$ and $b_{T2}$, are associated with deformation of the bonds along the Type 1 and Type 2 bond directions, respectively, as shown in Figure 2. All PD material constants can be expressed in terms of material constants of a cubic crystal, $Q_{ij}$, from classical theory. The procedure for obtaining these relationships is presented in Section 3.

When the stretch, $s_{\left(k)(j\right)}$ between material points *k* and *j* exceeds a critical stretch value, $s_{c}$, the interaction breaks and damage occurs. Hence, there will no longer be any interaction between these two particles. The critical stretch parameter (2D) can be expressed as [24]:

(11) $$s_{c}=\sqrt{\frac{G_{c}}{\left(\frac{6}{\pi }\mu +\frac{16}{9\pi^{2}}\left(\kappa -2\mu \right)\right)\delta }},$$

where *μ* represents the shear modulus and *κ* is the bulk modulus of the material. According to [42], the critical energy release rate $G_{c}$ can be obtained from fracture toughness $K_{Ic}$, as:

(12) $$\begin{array}{ccc} G_{c}=\frac{K_{Ic}^{2}}{E} & & \text{plane stress} \end{array},$$

where *E* is the Young’s modulus.

An “interface strength coefficient” is introduced by [20] to investigate various fracture modes of polycrystalline materials and is defined as:

(13) $$\beta =\frac{s_{c}^{GB}}{s_{c}^{GI}},$$

where $s_{c}^{GB}$ and $s_{c}^{GI}$ denote the critical stretch of interactions that cross the grain boundary and the critical stretch of interactions that are located within the grain, respectively, i.e., GB represents the grain boundary and GI represents the grain interior.

## 3. Derivation of PD Parameters

The PD material parameters, $a,d,b_{T1},\text{and}\;b_{T2}$, that appear in the force density vector-stretch relations for in-plane deformation of a cubic crystal can be related to the engineering constants by considering three different simple loading conditions:

1. Simple shear: $\gamma_{12}=\zeta$;
2. Uniaxial stretch in crystal orientation direction: $\varepsilon_{11}=\zeta ,\varepsilon_{22}=0$;
3. Biaxial stretch: $\varepsilon_{11}=\zeta ,\varepsilon_{22}=\zeta$.

### 3.1. First Loading Condition (Simple Shear $\gamma_{12}=\zeta$)

**Figure 3:**
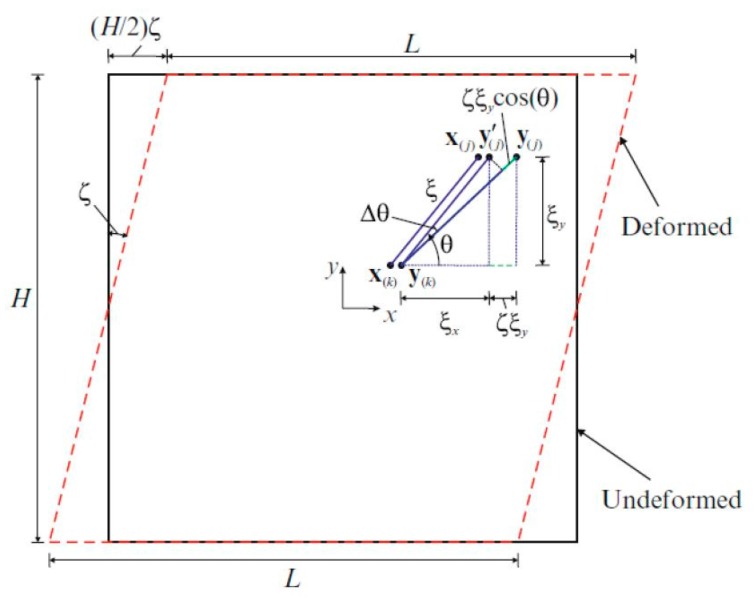
Simple shear loading condition (x: crystal orientation direction).

In the first loading condition, a constant simple shear strain is applied as shown in Figure 3 and the corresponding dilatation and strain energy density from classical continuum mechanics (CCM) can be expressed as:

(14) $$\theta_{\left(k\right)}^{CCM}=0$$

and

(15) $$W_{\left(k\right)}^{CCM}=\frac{1}{2}Q_{44}\zeta^{2}.$$

For this loading condition, the length of the relative position of material points $y_{\left(j\right)}$ and $y_{\left(k\right)}$ in the deformed state becomes:

(16) $$\left|y_{\left(j\right)}-y_{\left(k\right)}\right|=\left[1+\left(\text{sin}\;\phi_{\left(j\right)\left(k\right)}\text{cos}\;\phi_{\left(j\right)\left(k\right)}\right)\zeta \right]\left|x_{\left(j\right)}-x_{\left(k\right)}\right|.$$

The PD dilatation term can be evaluated as:

(17) $$\theta_{\left(k\right)}^{PD}=d\int_{H}\frac{\delta }{\xi }\left\{\left[1+\left(\text{sin}\;\phi \text{cos}\;\phi \right)\zeta \right]\xi -\xi \right\}dH=0,$$

in which $\xi =\left|x_{\left(j\right)}-x_{\left(k\right)}\right|$. As expected, there is no dilatation for this loading condition. Therefore, the strain energy density can be calculated as:

(18) $$\begin{array}{l} W_{\left(k\right)}^{PD}=a\left(0\right)+b_{T1}\int_{H}\frac{\delta }{\xi }{\left\{\left[1+\left(\text{sin}\;\phi \text{cos}\;\phi \right)\zeta \right]\xi -\xi \right\}}^{2}dH\\ +b_{T2}\sum_{j=1}^{J}\frac{\delta }{\left|x_{\left(j\right)}-x_{\left(k\right)}\right|}{\left(\left(\text{sin}\;\phi_{\left(j\right)\left(k\right)}\text{cos}\;\phi_{\left(j\right)\left(k\right)}\right)\zeta \left|x_{\left(j\right)}-x_{\left(k\right)}\right|\right)}^{2}V_{\left(j\right)} \end{array}$$

or

(19) $$W_{\left(k\right)}^{PD}=\left(\frac{\pi h\delta^{4}\zeta^{2}}{12}\right)b_{T1}+\left(\frac{\delta \zeta^{2}}{4}\sum_{j=1}^{J}\xi_{\left(j\right)\left(k\right)}V_{\left(j\right)}\right)b_{T2}.$$

Equating expressions of strain energy density from CCM and OSB PD formulations, i.e., Equations (15) and (19), results in:

(20) $$\left(\frac{\pi h\delta^{4}}{12}\right)b_{T1}+\left(\frac{\delta }{4}\sum_{j=1}^{J}\xi_{\left(j\right)\left(k\right)}V_{\left(j\right)}\right)b_{T2}=\frac{Q_{44}}{2}.$$

### 3.2. Second Loading Condition (Uniaxial Stretch in Crystal Orientation Direction: $\varepsilon_{11}=\zeta ,\varepsilon_{22}=0$)

**Figure 4:**
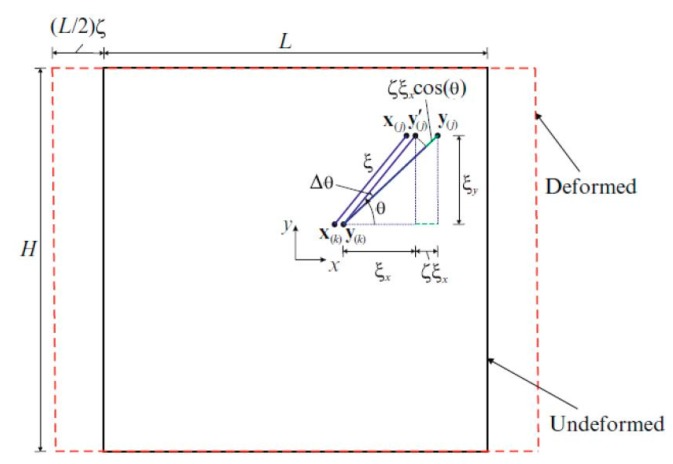
Uniaxial loading condition in crystal orientation direction, *x*.

In the second loading condition, a constant strain is applied along the direction of crystal orientation (Figure 4), and the corresponding dilatation and strain energy density from CCM can be expressed as:

(21) $$\theta_{\left(k\right)}^{CCM}=\zeta$$

and

(22) $$W_{\left(k\right)}^{CCM}=\frac{1}{2}Q_{11}\zeta^{2}.$$

The length of the relative position of material points $y_{\left(j\right)}$ and $y_{\left(k\right)}$ in the deformed state becomes:

(23) $$\left|y_{\left(j\right)}-y_{\left(k\right)}\right|=\left[1+\left({\text{cos}}^{2}\;\phi_{\left(j\right)\left(k\right)}\right)\zeta \right]\left|x_{\left(j\right)}-x_{\left(k\right)}\right|.$$

Due to this deformation, the dilatation of PD can be evaluated as:

(24) $$\theta_{\left(k\right)}^{PD}=d\int_{H}\frac{\delta }{\xi }\left\{\left[1+\left({\text{cos}}^{2}\;\phi \right)\zeta \right]\xi -\xi \right\}dH$$

or

(25) $$\theta_{\left(k\right)}^{PD}=\frac{\pi dh\delta^{3}\zeta }{2}.$$

By equating expressions of the dilatation term from CCM and PD formulations, i.e., Equations (21) and (25), results in:

(26) $$d=\frac{2}{\pi h\delta^{3}}.$$

The PD strain energy density for this loading condition can be calculated as:

(27) $$\begin{array}{l} W_{\left(k\right)}^{PD}=a\zeta^{2}+b_{T1}\int_{H}\frac{\delta }{\xi }{\left\{\left[1+\left({\text{cos}}^{2}\;\phi \right)\zeta \right]\xi -\xi \right\}}^{2}dH\\ +b_{T2}\sum_{j=1}^{J}\frac{\delta }{\left|x_{\left(j\right)}-x_{\left(k\right)}\right|}{\left(\left({\text{cos}}^{2}\;\phi_{\left(j\right)\left(k\right)}\right)\zeta \left|x_{\left(j\right)}-x_{\left(k\right)}\right|\right)}^{2}V_{\left(j\right)} \end{array}$$

or

(28) $$W_{\left(k\right)}^{PD}=a\zeta^{2}+\left(\frac{\pi h\delta^{4}\zeta^{2}}{4}\right)b_{T1}+\left(\frac{\delta \zeta^{2}}{4}\sum_{j=1}^{J}\xi_{\left(j\right)\left(k\right)}V_{\left(j\right)}\right)b_{T2}.$$

Hence, by equating expressions of strain energy density from Equations (22) and (28), the following relationship can be obtained:

(29) $$a+\left(\frac{\pi h\delta^{4}}{4}\right)b_{T1}+\left(\frac{\delta }{4}\sum_{j=1}^{J}\xi_{\left(j\right)\left(k\right)}V_{\left(j\right)}\right)b_{T2}=\frac{1}{2}Q_{11}.$$

### 3.3. Third Loading Condition (Biaxial Stretch: $\varepsilon_{11}=\zeta ,\varepsilon_{22}=\zeta$)

**Figure 5:**
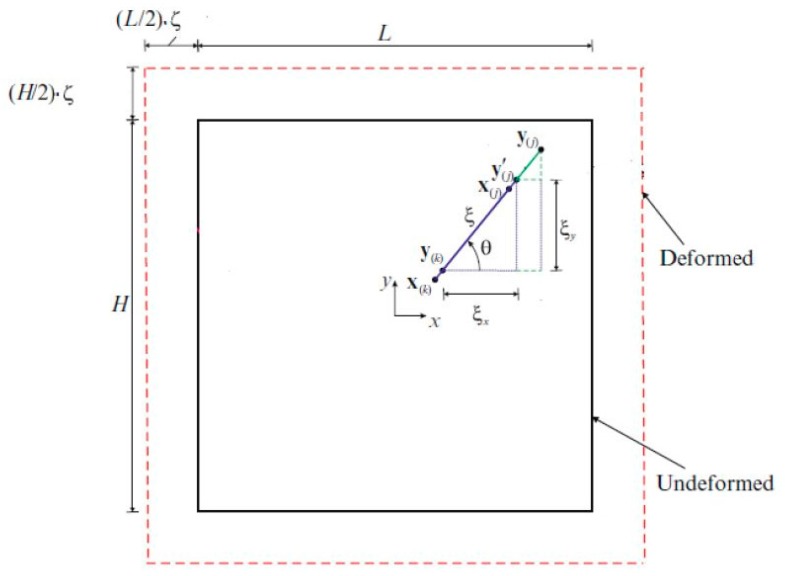
Biaxial stretch loading condition.

In the third loading condition, a constant strain is applied in all directions (Figure 5), and the corresponding dilatation and strain energy density from CCM can be expressed as:

(30) $$\theta_{\left(k\right)}^{CCM}=2\zeta$$

and

(31) $$W_{\left(k\right)}^{CCM}=\left(Q_{11}+Q_{12}\right)\zeta^{2}.$$

The length of the relative position under this loading condition can be evaluated as:

(32) $$\left|y_{\left(j\right)}-y_{\left(k\right)}\right|=\left[1+\left({\text{cos}}^{2}\;\phi_{\left(j\right)\left(k\right)}+{\text{sin}}^{2}\;\phi_{\left(j\right)\left(k\right)}\right)\zeta \right]\left|x_{\left(j\right)}-x_{\left(k\right)}\right|.$$

Hence, the dilatation term in PD formulation can be expressed as:

(33) $$\theta_{\left(k\right)}^{PD}=d\int_{H}\frac{\delta }{\xi }\left\{\left[1+\zeta \right]\xi -\xi \right\}dH$$

or

(34) $$\theta_{\left(k\right)}^{PD}=\pi dh\delta^{3}\zeta .$$

By equating the expressions of dilatation from both CCM and PD, i.e., Equations (30) and (34), the same expression given in Equation (26) can be obtained. Moreover, the PD strain energy density under this loading condition can be evaluated as:

(35) $$\begin{array}{l} W_{\left(k\right)}^{PD}=a\zeta^{2}+b_{T1}\int_{H}\frac{\delta }{\xi }{\left\{\left[1+\left({\text{sin}}^{2}\;\phi \right)\zeta \right]\xi -\xi \right\}}^{2}dH\\ +b_{T2}\delta \zeta^{2}\left(\sum_{j=1}^{J}\left|x_{\left(j\right)}-x_{\left(k\right)}\right|V_{\left(j\right)}\right) \end{array}.$$

By equating Equations (31) and (35), a new relationship can be obtained, as:

(36) $$Q_{11}+Q_{12}=4a+\left(\frac{2\pi h\delta^{4}}{3}\right)b_{T1}+\left(\delta \sum_{j=1}^{J}\xi_{\left(j\right)\left(k\right)}V_{\left(j\right)}\right)b_{T2}.$$

Hence, the OSB PD material parameters can be expressed in terms of engineering constants of CCM by utilizing the three relationships given in Equation (20), (29), and (36) as:

(37) $$\{\begin{array}{c} a=\frac{1}{2}\left(Q_{12}-Q_{44}\right)\\ b_{T1}=\frac{3\left(Q_{11}-Q_{12}\right)}{\pi h\delta^{4}}\\ b_{T2}=\frac{2Q_{44}-Q_{11}+Q_{12}}{\delta \left(\sum_{j=1}^{J}\left|x_{\left(j\right)}^{n}-x_{\left(k\right)}^{n}\right|V_{\left(j\right)}\right)}\\ d=\frac{2}{\pi h\delta^{3}} \end{array}.$$

For BB PD, the parameter *a* associated with dilatation should vanish, leading to the constraint equation $Q_{12}=Q_{44}$, which is a limitation of BB PD in cubic polycrystal analysis.

## 4. Numerical Results and Discussion

In this section, the results generated from static and dynamic PD analyses are presented, and comparisons with finite element method (FEM) results are also provided. For static analysis (Section 4.1), a single cubic Niobium (Nb) crystal model is considered first (Section 4.2.1) and displacement fields of PD and FEM are compared. Then, a cubic Molybdenum (Mo) polycrystal model with 18 Voronoi grains is analysed (Section 4.2.2), and the PD and FEM displacement fields are compared. For the dynamic analysis, the influence of the discretization size and the interface strength coefficient (*β*) on the results is considered first (Section 4.3.1). Then, the influence of crystal size on fracture behaviour is investigated (Section 4.3.2).

### 4.1. Material Data

Two different materials are considered in this study: niobium (Nb) for single crystal static analysis, and molybdenum (Mo) for polycrystal static and dynamic analysis. According to [43], the local stiffness matrix of each individual crystal can be written as:

(38) $$\left[C\right]=\left[\begin{array}{cccccc} C_{11} & C_{12} & C_{12} & 0 & 0 & 0\\ C_{12} & C_{11} & C_{12} & 0 & 0 & 0\\ C_{12} & C_{12} & C_{11} & 0 & 0 & 0\\ 0 & 0 & 0 & C_{44} & 0 & 0\\ 0 & 0 & 0 & 0 & C_{44} & 0\\ 0 & 0 & 0 & 0 & 0 & C_{44} \end{array}\right].$$

However, for plane stress configuration, the material matrix given in Equation (38) can be written by using reduced stiffness matrix following the procedure given in [44] as

(39) $$\left[Q\right]=\left[\begin{array}{ccc} Q_{11} & Q_{12} & 0\\ Q_{12} & Q_{11} & 0\\ 0 & 0 & Q_{44} \end{array}\right],$$

where $Q_{ij}$ are the reduced stiffness coefficients and can be expressed in terms of the elements of the stiffness matrix, $C_{ij}$ as

(40) $$\begin{array}{c} Q_{11}=\frac{{C_{11}}^{2}-{C_{12}}^{2}}{C_{11}}\\ \begin{array}{c} Q_{12}=\frac{C_{11}C_{12}-{C_{12}}^{2}}{C_{11}}\\ Q_{44}=C_{44} \end{array} \end{array}.$$

Therefore, the material properties of Nb and Mo are given in Table 1 as shown below [44]:

The fracture toughness of Mo is specified as $K_{IC}=24.2\;\text{MPa}\sqrt{m}$ [45], which corresponds to a critical stretch value of 0.008127 for plane stress configuration.

### 4.2. Static Analysis

**Figure 6:**
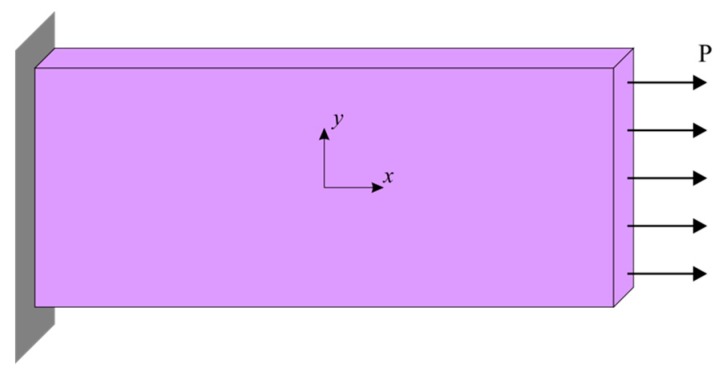
Crystal model for static analysis.

A cubic crystal model with a length of 152.4 μm and a width of 76.2 μm is considered and the number of particles along the horizontal and vertical directions is 240 and 120, respectively. The values of grid spacing and horizon are Δx=0.635 μm and δ=1.915 μm, respectively. A uniform discretization scheme is used throughout this study. However, non-uniform discretization can also be possible by using small grid sizes at critical regions such as interfaces and utilizing the “Dual-horizon peridynamics” concept, as introduced by Ren et al. (2016) [46,47]. A horizontal tension loading of, *P*, is applied on the right edge of the model, and the left edge is fully fixed as shown in Figure 6. The tension loading is specified as a body load and applied to a single layer of material points at the right edge of the model. The displacement constraint condition at the left edge is also imposed to a single layer of material points. To reach the steady-state condition and perform static analysis, an adaptive dynamic relaxation technique was utilized as described in Madenci and Oterkus (2014) [24].

#### 4.2.1. Static Analysis of Nb Single Crystal

**Figure 7:**
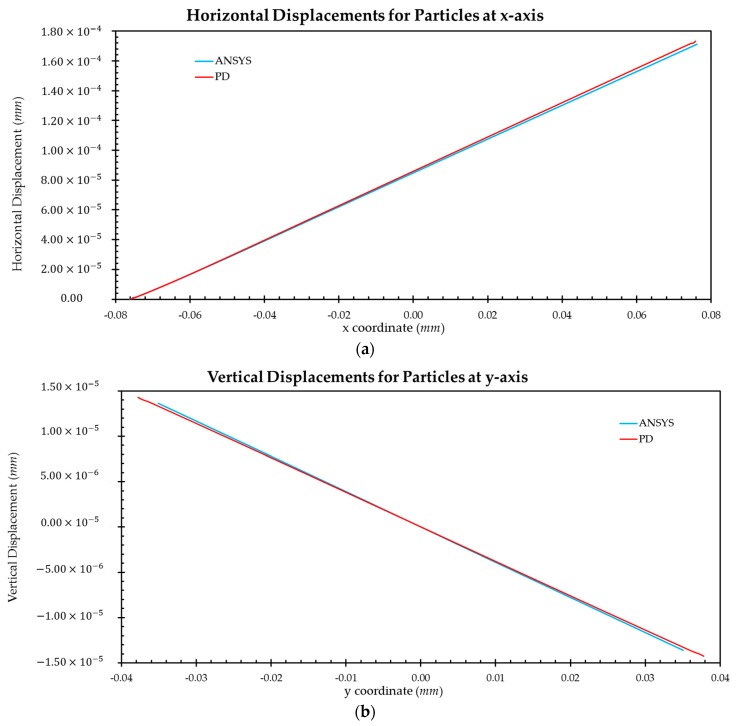
Comparison of displacements between FEM and PD analyses for Nb crystal for 0° orientation: (**a**) horizontal displacements for particles along the central x-axis; (**b**) vertical displacements for particles along the central y-axis.

**Figure 8:**
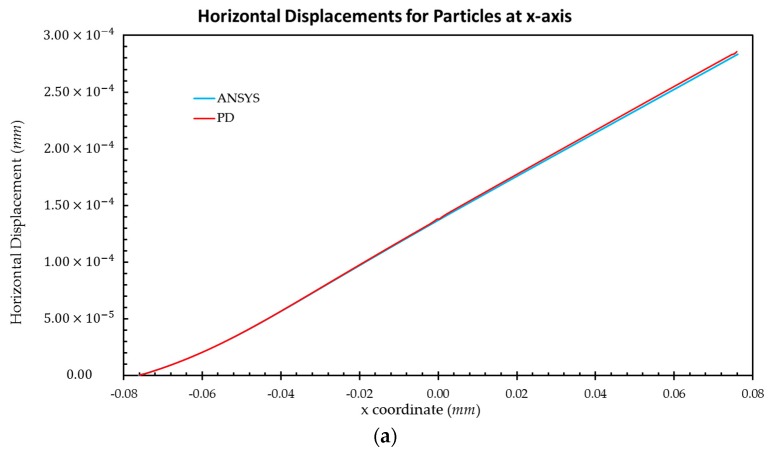

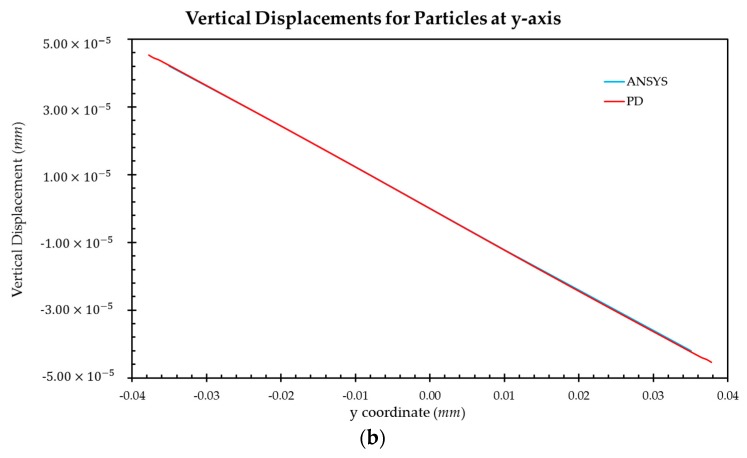
Comparison of displacements between FEM and PD analyses for Nb Crystal for 45° orientation: (**a**) horizontal displacements for particles along the central x-axis; (**b**) vertical displacements for particles along the central y-axis.

The tension loading applied on the right edge of the model is P=174.4 MPa. Figure 7 and Figure 8 show a comparison of the results obtained from FEM and PD analysis under plane stress conditions for crystal orientations of 0° and 45°, respectively. Particles located along the central x-axis and y-axis are selected and horizontal and vertical displacements are compared, respectively.

Based on the results presented in Figure 7 and Figure 8, good agreement is obtained between PD and FEM analyses. Therefore, it can be concluded that the OSB PD crystal model presented in this study can produce accurate results for different crystal orientations for a single crystal.

#### 4.2.2. Static Analysis of Mo Polycrystal

**Figure 9:**
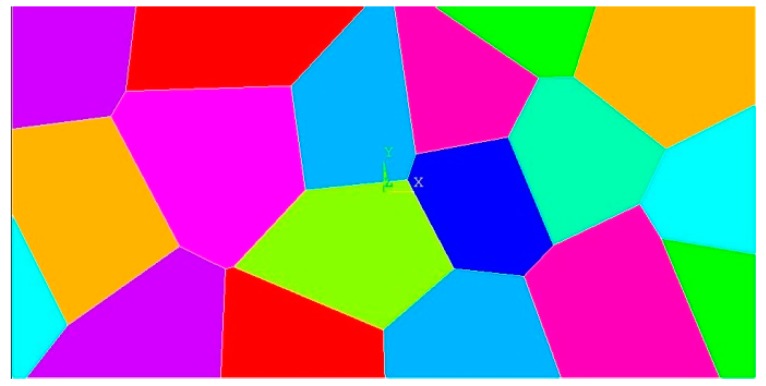
Model for the static analysis of Mo polycrystal, composed of 18 randomly orientated grains with respect to the x–y coordinate system located at the centre of the model.

In the second case, a polycrystal model with 18 randomly orientated grains is generated by using Voronoi tessellation. A uniform discretization is utilized. Depending on the location of the material point, corresponding grain orientation is determined. Hence, Type 2 bonds will exist in many different directions according to the random orientation of the crystals. The average crystal size is 645.16 μm^2^, and the amount of tension loading applied on the right edge is P=374.5 MPa. The layout of the polycrystal model is shown in Figure 9.

**Figure 10:**
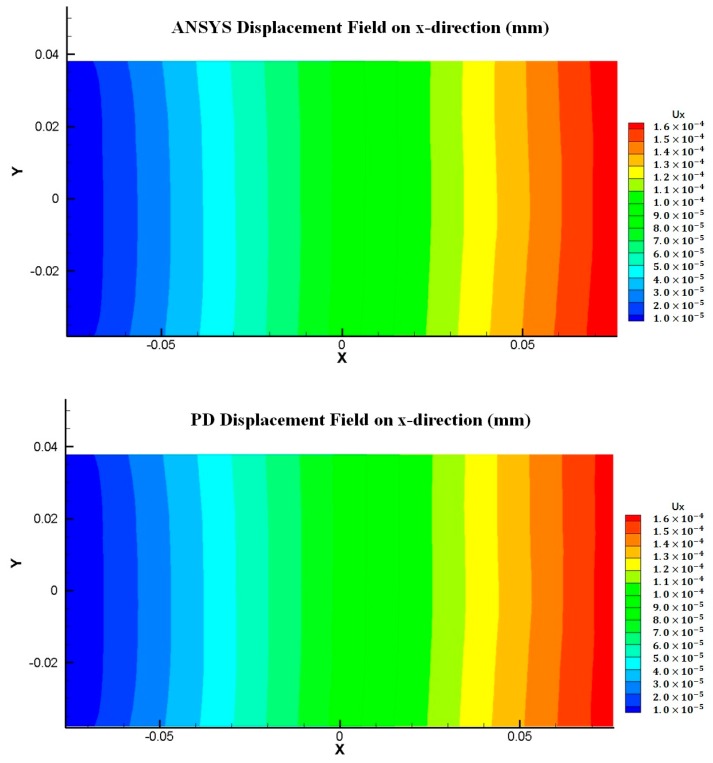
Displacement field comparison between FEM and PD analyses for Mo polycrystal (x-direction).

**Figure 11:**
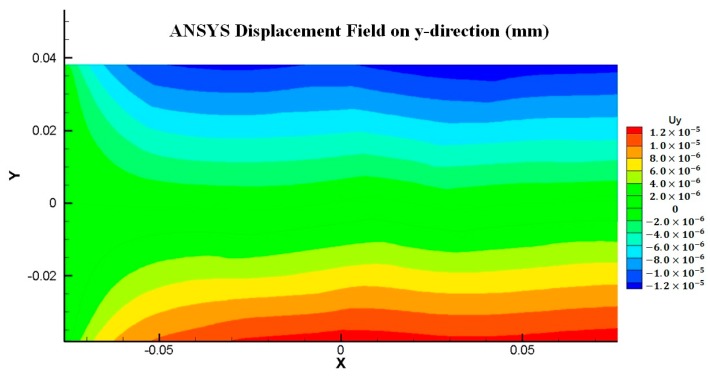

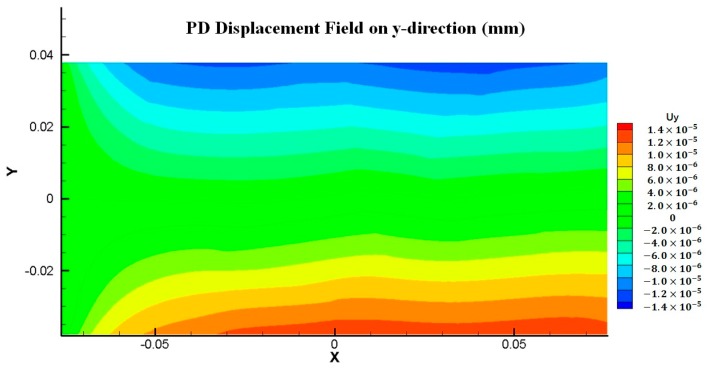
Displacement field comparison between FEM and PD analyses for Mo polycrystal (y-direction).

Displacement distributions for both x and y directions are compared between FEM and PD solutions as shown in Figure 10 and Figure 11 and a good agreement is obtained between two solutions, which confirms that the present model can also accurately represent polycrystal material behaviour.

### 4.3. Dynamic Analysis of Mo Polycrystals

**Figure 12:**
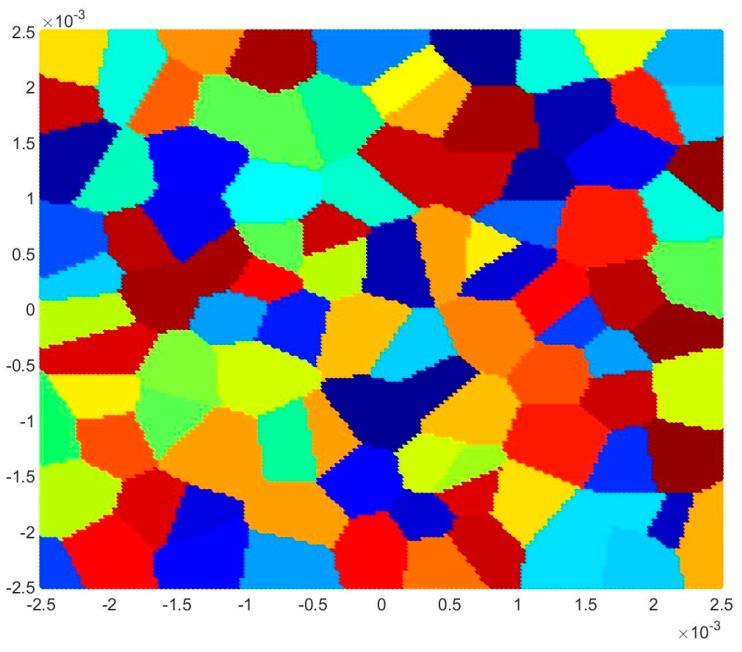
Polycrystal model for dynamic analysis (100 grains).

**Figure 13:**
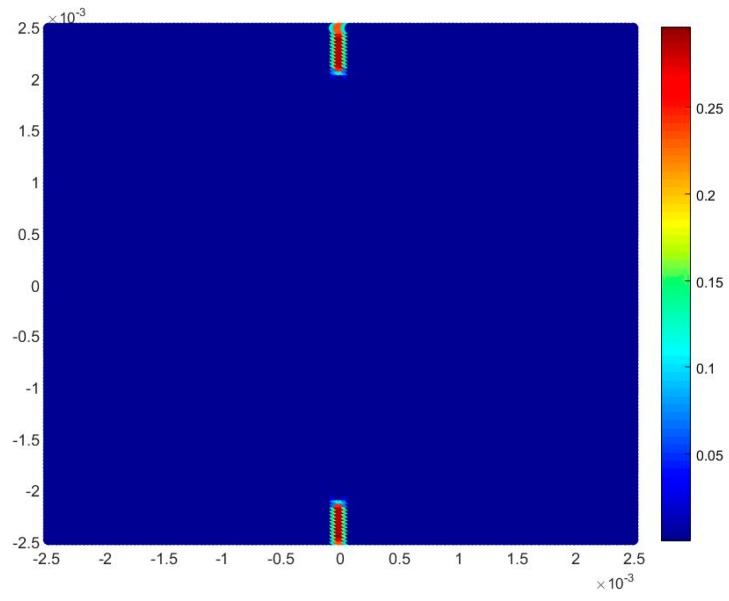
Location of the cracks in the model for dynamic analysis.

For the dynamic analysis, a 5 mm by 5 mm square plate with randomly oriented grains is considered as shown in Figure 12. A horizontal velocity boundary condition of V=5 m/s is applied on both the left and right edges of the model. Three layers of virtual particles are placed along the left and right edges to impose this condition, as suggested in [24]. A no-fail zone is also imposed on virtual particles and their neighbouring particles in order to allow the load to be accurately transferred inside the plate. Two pre-existing cracks with a length of 0.4 mm are applied at the centre of the bottom and top edges, as shown in Figure 13. The time step size is specified as dt=0.05 ns and the total number of time steps is 100,000, i.e., the total simulation time of 5.0 μs. The study considers three different interface strength coefficient, β, values (0.5, 1.0, and 2.0), three different mesh sizes (74 × 74, 150 × 150, and 300 × 300) and three different total numbers of grains (25, 100, and 400; i.e., different crystal sizes).

#### 4.3.1. Effect of PD Discretization Size and Interface Strength Coefficient (*β*)

The aim of this analysis is to investigate the effect of the peridynamic discretization size on the crack pattern predicted by PD model and the morphology of intergranular and transgranular fracture modes when changing the value of the interface strength coefficient, *β*. The horizon is specified as δ=3.015⋅Δx, which means that it is controlled by changing the PD discretization (74 × 74 particles, 150 × 150 particles, and 300 × 300 particles). Moreover, three different interface strength coefficient, *β*, values are considered to investigate the intergranular and transgranular fracture modes of the polycrystal.

**Figure 14:**
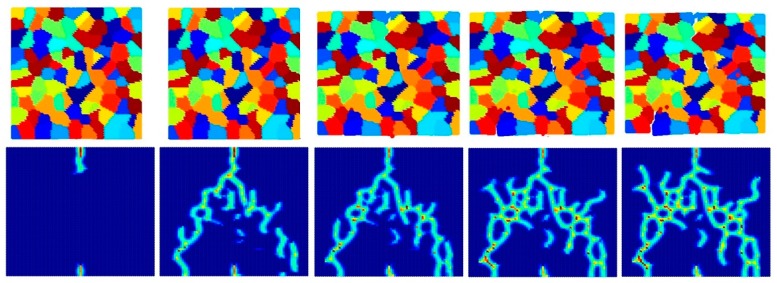
Fracture pattern of polycrystal when β=0.5 with 74 × 74 particles. From left to right: time = 1.5 μs, 2.0 μs, 2.5 μs, 3.0 μs, and 3.5 μs, respectively.

**Figure 15:**
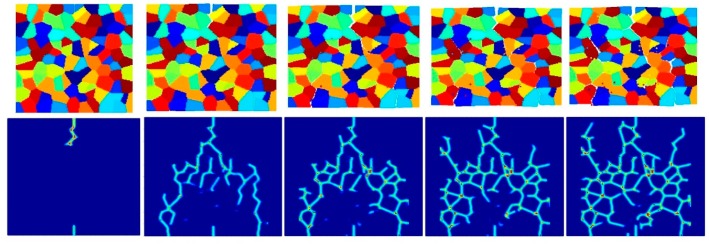
Fracture pattern of polycrystal when β=0.5 with 150 × 150 particles. From left to right: time = 1.5 μs, 2.0 μs, 2.5 μs, 3.0 μs, and 3.5 μs, respectively.

**Figure 16:**
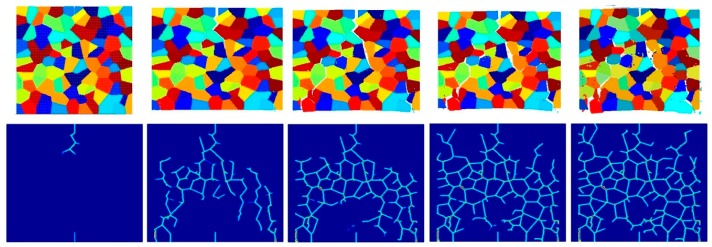
Fracture pattern of polycrystal when β=0.5 with 300 × 300 particles. From left to right: time = 1.5 μs, 2.0 μs, 2.5 μs, 3.0 μs, and 3.5 μs, respectively.

Figure 14, Figure 15 and Figure 16 show the fracture pattern of the polycrystal under plane stress configuration at five different times (1.5 μs, 2.0 μs, 2.5 μs, 3.0 μs, and 3.5 μs) for β=0.5 with 74 × 74 particles, 150 × 150 particles, and 300 × 300 particles, respectively.

The results show that with an increasing total number of particles, the intergranular crack pattern can be predicted more accurately and in more detail. However, the simulation time will increase rapidly as well. Therefore, it is important to find a good balance between accuracy and time. In this study, 150 × 150 particles can provide appropriate results, which is the reason why most of the simulations in this paper are chosen by using this number of particles.

**Figure 17:**
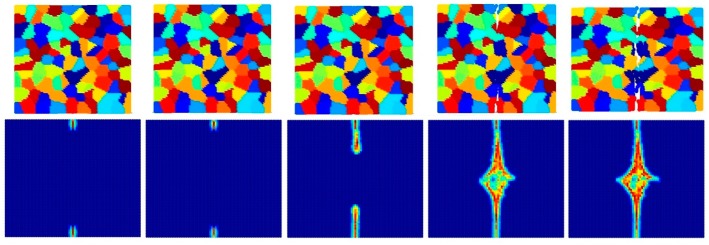
Fracture pattern of polycrystal when β=1.0 with 74 × 74 particles. From left to right: time = 1.5 μs, 2.0 μs, 2.5 μs, 3.0 μs, and 3.5 μs, respectively.

**Figure 18:**
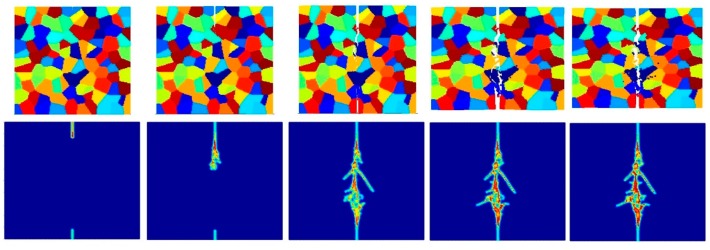
Fracture pattern of polycrystal when β=1.0 with 150 × 150 particles. From left to right: time = 1.5 μs, 2.0 μs, 2.5 μs, 3.0 μs, and 3.5 μs, respectively.

**Figure 19:**
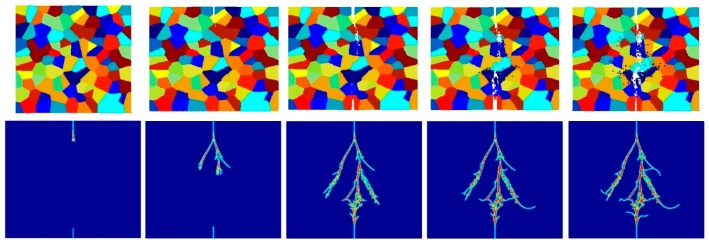
Fracture pattern of polycrystal when β=1.0 with 300 × 300 particles. From left to right: time = 1.5 μs, 2.0 μs, 2.5 μs, 3.0 μs, and 3.5 μs, respectively.

**Figure 20:**
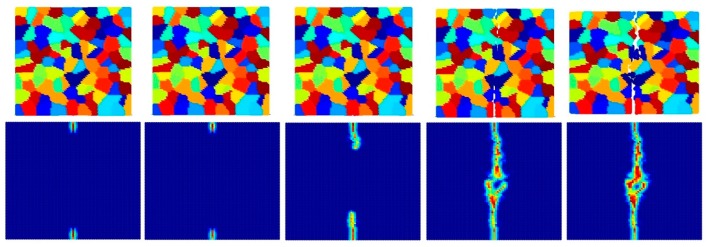
Fracture pattern of polycrystal when β=2.0 with 74 × 74 particles. From left to right: time = 1.5 μs, 2.0 μs, 2.5 μs, 3.0 μs, and 3.5 μs, respectively.

**Figure 21:**
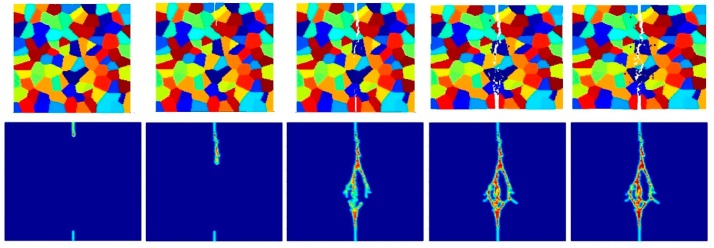
Fracture pattern of polycrystal when β=2.0 with 150 × 150 particles. From left to right: time = 1.5 μs, 2.0 μs, 2.5 μs, 3.0 μs, and 3.5 μs, respectively.

**Figure 22:**
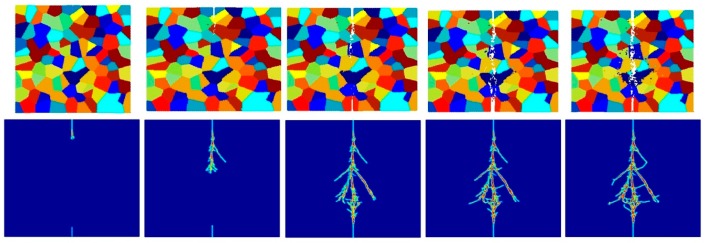
Fracture pattern of polycrystal when β=2.0 with 300 × 300 particles. From left to right: time = 1.5 μs, 2.0 μs, 2.5 μs, 3.0 μs, and 3.5 μs, respectively.

Figure 17, Figure 18, Figure 19, Figure 20, Figure 21 and Figure 22 show the fracture patterns of the polycrystal at five different times (1.5 μs, 2.0 μs, 2.5 μs, 3.0 μs, and 3.5 μs) for β=1.0 and β=2.0 with 74 × 74 particles, 150 × 150 particles, and 300 × 300 particles, respectively.

As described above, similar conclusions can be found in these simulations. For instance, branching of cracks can be obtained more clearly by increasing the total number of particles, but the simulations become more time-consuming. Moreover, the transgranular fracture mode becomes more dominant as the interface strength coefficient increases.

#### 4.3.2. Effect of the Crystal Size

The aim of this section is to investigate the effect of the crystal size on fracture pattern. The plate is discretized by 150 × 150 particles, containing three different numbers of randomly orientated grains (25 grains, 100 grains, and 400 grains). Three different grain boundary strength coefficients, β=0.5, β=1.0 and β=2.0, are considered to investigate the effect of crystal size for different fracture modes.

**Figure 23:**
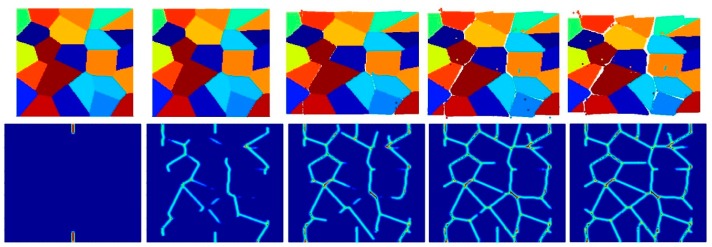
Fracture pattern of polycrystal when β=0.5 with 25 grains in total. From left to right: time = 1.5 μs, 2.0 μs, 2.5 μs, 3.0 μs, and 3.5 μs, respectively.

**Figure 24:**
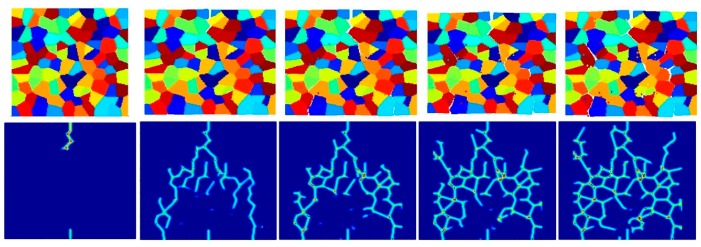
Fracture pattern of polycrystal when β=0.5 with 100 grains in total. From left to right: time = 1.5 μs, 2.0 μs, 2.5 μs, 3.0 μs, and 3.5 μs, respectively.

**Figure 25:**
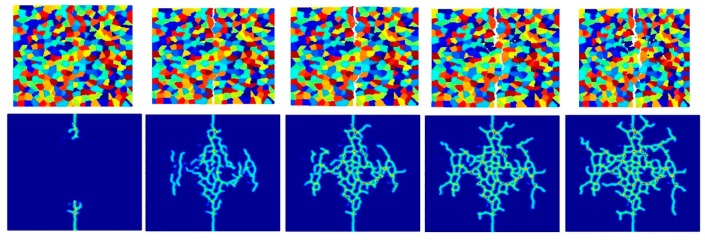
Fracture pattern of polycrystal when β=0.5 with 400 grains in total. From left to right: time = 1.5 μs, 2.0 μs, 2.5 μs, 3.0 μs, and 3.5 μs, respectively.

Figure 23, Figure 24 and Figure 25 show the fracture pattern of the polycrystal at five different times (1.5 μs, 2.0 μs, 2.5 μs, 3.0 μs, and 3.5 μs) for β=0.5 with 25 grains, 100 grains, and 400 grains, respectively.

**Figure 26:**
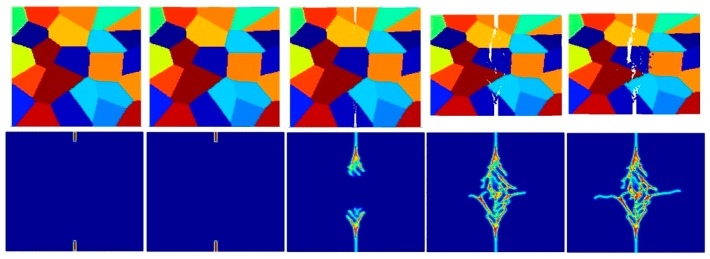
Fracture pattern of polycrystal when β=1.0 with 25 grains in total. From left to right: time = 1.5 μs, 2.0 μs, 2.5 μs, 3.0 μs, and 3.5 μs, respectively.

**Figure 27:**
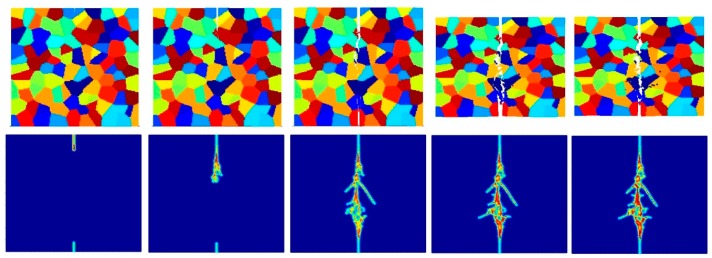
Fracture pattern of polycrystal when β=1.0 with 100 grains in total. From left to right: time = 1.5 μs, 2.0 μs, 2.5 μs, 3.0 μs, and 3.5 μs, respectively.

**Figure 28:**
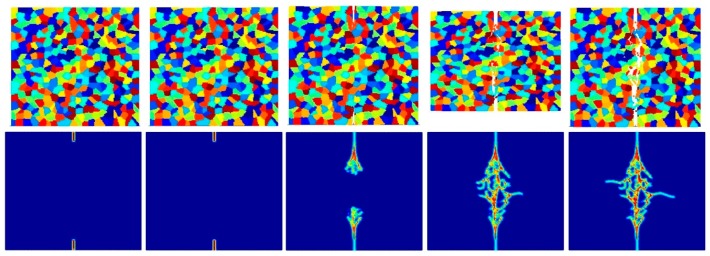
Fracture pattern of polycrystal when β=1.0 with 400 grains in total. From left to right: time = 1.5 μs, 2.0 μs, 2.5 μs, 3.0 μs, and 3.5 μs, respectively.

**Figure 29:**
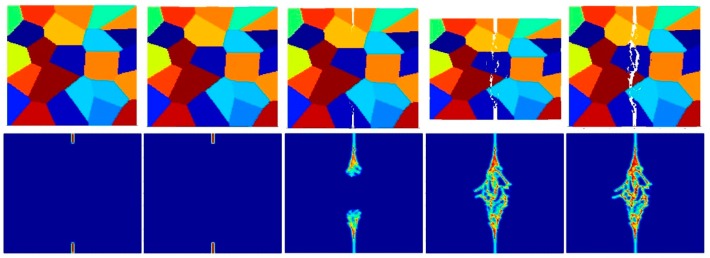
Fracture pattern of polycrystal when β=2.0 with 25 grains in total. From left to right: time = 1.5 μs, 2.0 μs, 2.5 μs, 3.0 μs, and 3.5 μs, respectively.

**Figure 30:**
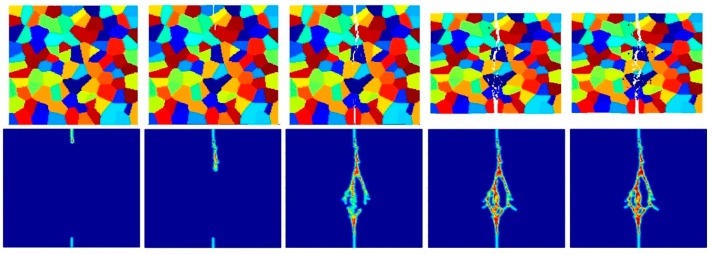
Fracture pattern of polycrystal when β=2.0 with 100 grains in total. From left to right: time = 1.5 μs, 2.0 μs, 2.5 μs, 3.0 μs, and 3.5 μs, respectively.

**Figure 31:**
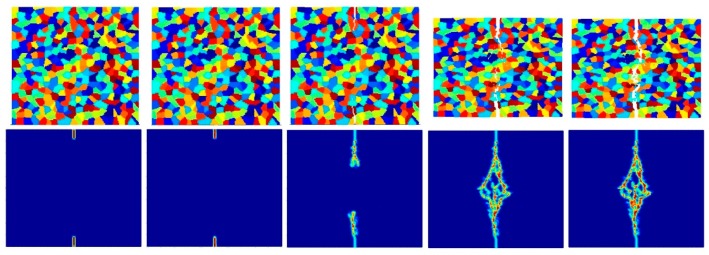
Fracture pattern of polycrystal when β=2.0 with 400 grains in total. From left to right: time = 1.5 μs, 2.0 μs, 2.5 μs, 3.0 μs, and 3.5 μs, respectively.

According to the damage plots shown in Figure 23 with 25 grains at 2.0 μs, the propagation does not always occur from pre-existing cracks. Only the top pre-existing crack propagates in the 100-grain model and both pre-existing cracks propagate in the 400-grain model. This is because with an increase in the total number of grains, the probability of the pre-existing cracks being located on a grain boundary increases. In other words, since the grain boundary strength β=0.5 promotes intergranular fracture mode, the crack can more easily propagate if it is located on the grain boundary. However, for the grain boundary strength values of β=1.0 and β=2.0, there is no such difference observed in fracture behaviour and both pre-existing cracks propagate as shown in Figure 26, Figure 27, Figure 28, Figure 29, Figure 30 and Figure 31.

## 5. Conclusions

In this paper, a new ordinary state-based peridynamic formulation is presented and related derivations are provided. The current model does not have any limitations on material constants as in the bond-based peridynamic theory. Static analyses were carried out for validation purposes and a comparison of results between PD and FEM shows that the proposed PD model can accurately capture the deformation behaviour of cubic polycrystalline materials. Then, dynamic analyses were carried out by considering different configurations to investigate the effect of interface strength coefficient, discretization size, and crystal size. The observations based on the evaluated results can be summarized as:

1. Intergranular and transgranular fracture modes can be captured by changing the interface strength coefficient. As a future study, by comparing the experimental and PD results of crack morphology, actual interface strength coefficients can be estimated.
2. The accuracy of simulation can be improved by increasing the total number of particles for intergranular fracture. However, the difference is not significant for transgranular fracture. In order to prevent the simulation from being time-consuming, a good balance should be considered between accuracy and simulation time.
3. Pre-existing cracks can propagate more easily with decreasing crystal size for inter-granular fracture mode, since there is a higher probability of a pre-existing crack interacting with a grain boundary.

As a future study, experimental studies can be used to validate and refine the damage predictions of the proposed PD model. Moreover, as the current study is mainly focused on a 2D model, the formulation can be extended to a 3D model.

## Figures and Tables

**Table 1:** Material properties of niobium and molybdenum.

Niobium				Molybdenum			
C_11_	239.8 GPa	Q_11_	174.4 GPa	C_11_	441.6 GPa	Q_11_	374.5 GPa
C_12_	125.2 GPa	Q_12_	59.82 GPa	C_12_	172.7 GPa	Q_12_	105.4 GPa
C_44_	28.22 GPa	Q_44_	28.22 GPa	C_44_	121.9 GPa	Q_44_	121.9 GPa

## Nomenclature

CZM	cohesive zone model
FEM	finite element method
CCM	classical continuum mechanics
PD	peridynamics
BB	bond-based
OSB	ordinary state-based
NOSB	non-ordinary state-based
$H_{x}$	horizon of a generic particle x
δ	radius of the horizon [m]
f	mechanical response function [N/m^6^]
c	bond constant [N/m^6^]
s	bond stretch
$s_{0}$	critical stretch
x	vector defining the position of a generic particle x
$x^{\prime }$	vector defining the position of a generic neighbour of particle x
y	vector defining the position of particle x in the deformed configuration
$y^{\prime }$	vector defining the position of particle $x^{\prime }$ in the deformed configuration
b(x,t)	body force density field [**N/m^3^**]
$K_{IC}$	fracture toughness $\left[\text{MPa}\sqrt{m}\right]$
h	plate’s thickness [**m**]
*κ*	bulk modulus [**N/m^2^**]
μ	shear modulus [**N/m^2^**]
$G_{c}$	critical energy release rate [**N/m**]
[C]	local stiffness matrix
[Q]	reduced stiffness matrix
Δx	grid spacing [**m**]
$b_{T1}$	bond constant type-1 [**N/m^6^**]
$b_{T2}$	bond constant type-2 [**N/m^6^**]
$\xi_{ij}$	undeformed bond length between particles i and j [**m**]
*ϕ*	bond angle with respect to the crystal orientation angle [**rad**]
$V_{j}$	volume of a generic neighbouring particle j [**m^3^**]
u(x,t)	displacement field at x [**m**]
$u\left(x^{\prime },t\right)$	displacement field at $x^{\prime }$ [**m**]
ρ(x)	mass density at x [**Kg/m^3^**]
$\ddot{u}\left(x,t\right)$	acceleration vector field [**m/s^2^**]
*β*	interface strength coefficient
